# Unraveling the connections between gut microbiota, stress, and quality of life for holistic care in newly diagnosed breast cancer patients

**DOI:** 10.1038/s41598-023-45123-1

**Published:** 2023-10-20

**Authors:** Chi-Chan Lee, Horng-Woei Yang, Chih-Ju Liu, Fang Lee, Wen-Ching Ko, Yuan-Ching Chang, Po-Sheng Yang

**Affiliations:** 1 Department of General Surgery, MacKay Memorial Hospital, Taipei, Taiwan; 2 Department of Medicine, MacKay Medical College, New Taipei, Taiwan; 3 Division of Molecular Medicine, Department of Medical Research, MacKay Memorial Hospital, Taipei, Taiwan; 4 Department of Nursing, MacKay Memorial Hospital, Taipei, Taiwan; 5 Department of Nursing, MacKay Medical College, New Taipei, Taiwan

**Keywords:** Cancer, Microbiology, Psychology, Health care, Medical research, Oncology

## Abstract

There is little research about the stress, quality of life (QOL) and gut microbiota in newly diagnosed breast cancer patients. In this study addressing the dearth of research on stress, quality of life (QOL), and gut microbiota in newly diagnosed breast cancer patients, 82 individuals were prospectively observed. Utilizing the Functional Assessment of Chronic Illness Therapy (FACT)-Breast questionnaire to assess health-related quality of life (HRQOL) and the Distress Thermometer (DT) to gauge distress levels, the findings revealed a mean FACT-B score of 104.5, underscoring HRQOL's varied impact. Significantly, 53.7% reported moderate to severe distress, with a mean DT score of 4.43. Further exploration uncovered compelling links between distress levels, FACT-B domains, and microbial composition. Notably, *Alcaligenaceae* and *Sutterella* were more abundant in individuals with higher DT scores at the family and genus levels (p = 0.017), while *Streptococcaceae* at the family level and *Streptococcus* at the genus level were prevalent in those with lower DT scores (p = 0.028 and p = 0.023, respectively). This study illuminates the intricate interplay of stress, QOL, and gut microbiota in newly diagnosed breast cancer patients, offering valuable insights for potential interventions of biomarker or probiotics aimed at alleviating stress and enhancing QOL in this patient cohort.

## Introduction

Female breast cancer has surpassed lung cancer as the most common diagnosed cancer, with an estimated 2.3 million new cases (11.7%), and with 68.5 thousand deaths^1^. Due to the early screening and improvement in the diagnosis and treatment, breast cancer patients’ survival outcomes including disease-free survival and overall survival improved progressively^2^. This improvement has also led to the need for more intensive management in terms of psychological issues such as quality of life (QOL), stress, anxiety and depression in breast cancer patients. The anxiety and depression negatively affect the quality of life and survival rates in breast cancer patients^3^.

The human microbiota is the collection of microbes that inhabit various parts of the body, primarily the gut, skin, vagina, mouth, and among others. Each body site has a distinct microbiota and there is significant inter-individual variability in microbiomes, which can contribute to diseases such as metabolic disorders, inflammatory diseases, allergies, and cancer^4–6^. The gut microbiota appears to influence breast cancer risk, response to treatment, and recurrence by affecting human health through metabolic, neural, and endocrine signaling, and immune activity^7^. The gut microbiota dysbiosis (imbalance) may lead to the development of breast cancer through the crosstalk among microbiota and both endogenous hormones and estrogen-like compounds might synergize to provide protection from disease but also to increase the risk of developing hormone-related diseases^8–11^. Besides, diversity and specific microbiota were linked to chemotherapy response as well as prognosis in breast cancer patients^12^. Microbiota diversity was also predictive of side effects such as neurological symptoms, weight gain, and constipation. Emerging evidence indicates that gut microbiota affects the response to anticancer therapies by modulating the host immune system and gut microbiota involvement in trastuzumab efficacy represents the foundation for new therapeutic strategies aimed at manipulating commensal bacteria to improve response in trastuzumab-resistant breast cancer patients^13^. Fu et al. found that depletion of intratumor bacteria significantly reduced lung metastasis without affecting primary tumor growth, offering new methods for improved breast cancer management^14^. Furthermore, modulating microbiota by nutritional treatment with probiotics and prebiotics is as emerging and promising strategies for prevention and treatment of breast cancer in the future^15^.

Many of the breast cancer patients experience fatigue, depression, and/or anxiety months to years after their breast cancer diagnosis with these symptoms being associated with greater disability and a poorer quality of life^16^. The gut microbiome plays a role between stress response, inflammation, and depression, and anxiety through the microbiome-gut-brain axis, which plays a key role in the regulation of brain function and behavior^17–19^. Recent study revealed gut bacteria composition may play a role in depression through the production of neurotransmitters, such as serotonin and glutamate^20^. A meta-analysis of 34 controlled clinical trials found that probiotics showed small but significant benefits for depression and anxiety, while prebiotics did not differ from placebo in their effects on depression or anxiety^21^. Additional randomized clinical trials with psychiatric samples are necessary fully to evaluate their therapeutic potential. There is little research about the quality of life^22^, stress, and gut microbiota in newly diagnosed breast cancer patients, so we designed this prospective study hoping to find potential probiotics for decreasing stress and improving quality of life in breast cancer patients.

## Methods

### Patient population

The study was designed as a prospective observational research project and was approved by the Institutional Review Board of MacKay Memorial Hospital (MMH), Taipei, Taiwan (19MMHIS061e). The breast cancer patients with stage I-IV who were diagnosed by core biopsy and age greater than 20 years old were included. All patients were treated in MMH and provided written informed consent. Recurrent breast cancer patients or those patients with a history of mental illness were excluded. The patients were recruited as convenience samples. The quality of life was evaluated by FACT-Breast questionnaire^23^ (supplement Table 1). The distress scale was evaluated by Distress Thermometer (Chinese version)^24,25^. All the fecal sample collection and FACT-Breast questionnaire and Distress Thermometer evaluation were performed on the first admission for breast cancer treatment. Every patient also completed a lifestyle habits survey, which included questions about alcohol consumption, use of gastroenterology medications and antibiotics, bowel habits, presence of blood in stools, and history of gastrointestinal conditions such as gastroenteritis, irritable bowel syndrome, chronic diarrhea of constipation and colon polyps.

### Quality of life and stress evaluation methods

#### Functional assessment of chronic illness therapy (FACT)-breast questionnaire

The Functional Assessment of Chronic Illness Therapy (FACT)-Breast is a health-related quality of life (HRQOL) questionnaire specifically designed to assess the impact of breast cancer on an individual's daily functioning^23^. The FACT-Breast questionnaire consists of a set of questions that measure different aspects of HRQOL such as physical, emotional, and social functioning, as well as overall well-being. The FACT-B consists of two parts, including the FACT-General (FACT-G) with 27 questions and the Breast Cancer Supplement (BCS) with 10 questions. It uses a 5-point scoring system, where 0 represents no at all and 4 represents a lot. The FACT-G includes four sub-scales: Physical Well-Being (PWB) with 7 questions, Social/Family Well-Being (SWB) with 7 questions, Emotional Well-Being (EWB) with 6 questions, and Functional Well-Being (FWB) with 7 questions. The BCS domain includes additional specific items about breast cancer: physical, psychological and aesthetical disorders due to cancer and therapies. The score of the FACT-B is the total of all life quality scores, with a higher score indicating higher satisfaction with life quality. The FACT-Breast questionnaire is widely used in research and clinical practice and has been shown to have good reliability and validity and the FACT-B in the Chinese version were confirmed^26^.

#### Distress thermometer

The Distress Thermometer^24,25^ is a single-item, 11-point visual analogue scale, with respondents indicating how distressed they have felt over the past week (from “No Distress” to “Extreme Distress”). The most recent version of the NCCN practice guidelines for the management of distress recommends that a DT score of 5 or higher indicates moderate-to-severe distress. It is a simple, self-report, pencil-and-paper measure, using a thermometer format line to rate the level of distress and is an accurate, valid screening tool for depression, anxiety^27^.

### Fecal samples collection and DNA extraction of microbiota

Each fecal samples were collected before treatment when breast cancer confirmed by core biopsy and store in − 20 °C refrigerator before use. DNA extraction from fecal samples using QIAamp Fast DNA stool mini kit (QIAGEN GmbH, Hilden Germany) followed by user’s manual. Briefly, 0.2 g sample in 1 mL InhibitEX buffer with glass beads, homogenized by precellys homogenizer (Bertin Instruments, Montigny-le-Bretonneux France) 4500 beat per min, 2 min. Heat the suspension for 10 min at 70 °C centrifuge sample for 1 min to pellet stool particles. 25 uL proteinase K in a new 2 mL centrifuge tube add 600 uL supernatant from stool pellet. Then add 600 AL buffer and mix-well. Incubate at 70 °C for 10 min. Add 600 uL 100% ethanol and mix-well. Filtrate sample by QIAamp spin column 13,000 *rpm*, 1 min. Wash filter by AW1, AW2 buffer. Elute DNA sample by 100 uL ATE buffer. DNA amount and quality was measured by nanodrop 2000 (Thermo Scientific, MA USA).

### 16S rRNA library construction and sequencing

Variable regions of 16S rRNA are frequently used in phylogenetic classifications such as genus or species in diverse microbial populations. 2.5 μl (50 ng) of DNA was used to set up the first PCR with 0.2 μM V3 + V4 forward and reverse primers (Forward:TCGTCGGCAGCGTCAGATGTGTATAAGAGACAGCCTACGGGNGGCWGCAG, Reverse: GTCTCGTGGGCTCGGAGATGTGTATAAGAGACAGGACTACHVGGGTATCTAATCC) and 12.5 μl 2X Kapa HiFi HotStart ReadyMix (KapaBiosystems) in 25 μl reactions. The PCR cycling conditions were 3 min at 95 °C, 25 cycles of 30 s at 95 °C, 30 s at 55 °C and 30 s at 72 °C, followed by 5 min at 72 °C. The amplified DNA was purified with Agencourt AMPure XP Reagent beads (Beckman Coulter Inc., CA, USA). The second PCR was set up to add indexes to the amplified DNA by adding 5 μl of purified DNA to 25 μl 2X Kapa HiFi HotStart ReadyMix (KapaBiosystems, MA, USA), 5 μl Nextera XT Index 1 and 2 primers (Illumina, CA, USA) in 50 μl reactions. The reaction was set at 3 min at 95 °C, 8 cycles of 30 s at 95 °C, 30 s at 55 °C and 30 s at 72 °C, followed by 5 min at 72 °C on an Applied Biosystems 2720 thermocycler (Thermo Fisher Scientific, CA, USA), followed by another Agencourt AMPure XP Reagent beads purification (Beckman Coulter Inc., CA, USA).

We used qPCR (KAPA SYBR FAST qPCR Master Mix) to quantify each library using Roche LightCycler 480 system and pooled then equally to 4nM for illumina MiSeq NGS system (illumina, CA, USA). More than 80,000 reads with paired-end sequencing (2*300bp) were generated, and the metagenomics workflow classified organisms from the amplicon using a database of 16S rRNA data (https://www.basespace.illumina.com). The classification was based on the NCBI database (https://www.ncbi.nlm.nih.gov). The output of the workflow was a classification of reads at several taxonomic levels: kingdom, phylum, class, order, family, genus, and species. Then the sequences were analyzed using the QIIME2 software package version 2017.10 (https://qiime2.org/). Potential chimeric sequences were removed using DADA2^28^, followed by trimming 30 and 90 bases of the 3′ region of the forward and the reverse reads, respectively. Taxonomical classification was performed using Naive Bayes classifier trained on the Greengenes13.8 with a 99% threshold of OTU full-length sequences.

### Statistical analysis

All data are presented as the means ± SD. Student's t‐test was used for comparison between two groups. One‐way ANOVA or two‐way ANOVA was performed for comparisons between multiple groups. Statistical analyses were performed using SPSS 26.0 software. A p‐value < 0.05 was considered statistically significant.

We presented bacterial compositions at the Family, Genus and Species levels and calculated the alpha, beta-diversity indices by MicrobiomeAnalyst (https://www.microbiomeanalyst.ca)^29,30^. Alpha-diversity is measured within a single sample using Shannon index with the QIIME software package version 2017.10 (https://qiime2.org/)^31^. For genera with a median relative abundance exceeding 1%, we conducted multiple regression analysis with adjustment for confounders to examine the association between FCR and the bacterial composition. We excluded from the analysis bacterial taxa that were not detected in 5% or more of the final participants. Furthermore, we calculated the skewness and kurtosis of the bacterial compositions and transformed the distribution of any bacterial compositions that did not assume a normal distribution using Box-Cox transformation. A p-value and T-test are calculated for each genus and species to assess statistical significance. Beta-diversity is measured between different samples using the Bray–Curtis index, and PCoA is used to visualize the results. PERMANOVA is used to test the significance of differences between samples, and F-value, r-squared, and p-value are reported. The taxonomy labels using QIIME. The stacked plot shows the percentage abundance (PA) of different genera and species in each sample, and the Top 20 genera and species are presented in separate graphs to highlight the most abundant taxa. Linear discriminant analysis (LDA) effect size (LEfSe) is a statistical method used to identify features that are differentially abundant between groups of samples. It calculates a p-value and Log LDA score for each feature and reports the results in a graphical format. The original sample pool is divided into Family, Genus, and Species categories, and features with a p-value < 0.05 and Log LDA score > 1.0 are considered significant.

## Results

### Patients characteristics

From May 2019 to May 2022, total 82 female breast cancer patients proved by core biopsy were included in this study. All 82 patients had fecal sample collection and FACT-Breast questionnaire and Distress Thermometer evaluation on the first admission for breast cancer treatment prospectively. The age ranged from 30 to 75 years old (average 45.7 years old). As in supplement Table 2, most of the patients had early-stage disease, including 2 (2.4%) with stage 0, 19 (23.2%) with stage I, 48 (58.5%) with stage II, 9 (11.0%) with stage III, and 4 (4.9%) with stage IV. All patients except stage IV cases (total 78 cases) received breast operation. Chemotherapy was applied in 71 out of 82 patients (86.6%) and radiotherapy in 34 out of 82 patients (41.5%). Total 49 patients (59.8% of 82 patients) received hormone therapy in this series.

### QOL of newly diagnosed breast cancer patients evaluated by FACT-B

Descriptive statistics for FACT-B different domain scores at diagnosis of breast cancer patients are shown in Table 1. The mean score of the FACT-B was 104.5 (SD, 19.76).

**Table 1:** FACT-B scores at diagnosis of breast cancer patients (n = 82).

	Minimal	Maximal	Mean	SD	Score range
Physical well being (PWB)	13	28	23.8	3.28	0–28
Social/Family well being (SWB)	0	28	19.5	6.09	0–28
Emotional well being (EWB)	6	108	18.6	10.72	0–24
Functional well-being (FWB)	2	28	18.5	5.89	0–28
Breast cancer subscale (BCS)	11	35	24.1	5.24	0–40
TOI	40	87	66.4	11.44	0–96
FACT-G	46	177	80.4	17.30	0–108
FACT-B	68	201	104.5	19.76	0–148

*TOI* FACT-B trial outcome index, (PWB score) + (FWB score) + (BCS score) = FACT-B TOI; FACT-G Total score = (PWB score) + (SWB score) + (EWB score) + (FWB score); FACT-B total score = (PWB score) + (SWB score) + (EWB score) + (FWB score) + (BCS score) (Please refer to supplement Table 1), *SD* standard deviation.

### Patients endorsing variable on distress thermometer

An initial DT was completed by all 82 patients. The mean score was 4.43 (range 0–10), with 53.7% (44/82) of the patients reporting moderate to severe distress (score 5 or above). Table 2 presents the problems indicated at presentation. Practical concerns (72% of patients) and emotional concerns (62.2% of patients) are the most sources of distress that can be identified by patients using the Distress Thermometer (DT). The most prevalent problem indicated at presentation was the treatment decisions in 51.2% (42/82) patients. In the emotional category, the nervousness and worry presented in more than 30% of the patients.

**Table 2:** Percentage of at diagnosis of breast cancer patients endorsing variable on Distress Thermometer problem list (n = 82).

	Problem list	No	Yes (%)
**Practical concerns**	**Practical concerns**	59	72.0
	Child care	12	14.6
	Housing	3	3.7
	Insurance/Finances	7	8.5
	Transportation	3	3.7
	Work/School	13	15.9
	Treatment decisions	42	51.2
**Family concerns**	Family concerns	17	20.7
	Dealing with children	6	7.3
	Dealing with spouse or partner	2	2.4
	Ability to have children	0	0.0
	Family health issues	8	9.8
**Spiritual or religious concerns**	Spiritual or religious concerns	8	9.8
**Physical concerns**	Physical concerns	40	48.8
	Changes in appearance	7	8.5
	Taking care of myself	2	2.4
	Constipation/Diarrhea	18	22.0
	Memory or concentration	3	3.7
	Nose dry/congested	4	4.9
	Pain	14	17.1
	Sexual health	3	3.7
	Skin dry/itching	3	3.7
	Sleep	15	18.3
	Tingling in hands/feet	4	4.9
	Fatigue	6	7.3
**Emotional concerns**	Emotional concerns	51	62.2
	Depression	12	14.6
	Fear	11	13.4
	Nervousness	34	41.5
	Sadness	15	18.3
	Worry	31	37.8
	Loss of interest or enjoyment	4	4.9

### Comparison of DT scores with FACT-B, stage, treatment modality and lifestyle variants

To determine the risk factors of DT score, we compared the DT score (DT score of 5 or higher indicates moderate-to-severe distress) with FACT-B subscale scores, stage, treatment modality and lifestyle variants separately. There is no difference of DT score in FACT-B subscale scores (Table 3), stage, treatment modality (supplement Table 2) and lifestyle variants (supplement Table 3) separately in our newly diagnosed breast cancer patients. But age and education status had the significantly different. Patients with age less than 50 years old and education above bachelor’s degree had higher DT score in our series.

**Table 3:** Comparison the DT and FACT-B subclass assessment dimensions.

Dimension	DT score	No	Mean	S.D	*t*
PBW	0–4	38	24.08	3.283	0.606
	5–10	44	23.64	3.307	
SWB	0–4	38	20.37	5.782	1.221
	5–10	44	18.73	6.307	
EWB	0–4	38	18.21	3.757	− 0.273
	5–10	44	18.86	14.292	
FWB	0–4	38	19.21	5.818	0.998
	5–10	44	17.91	5.953	
BCS	0–4	38	24.66	5.148	0.919
	5–10	44	23.59	5.324	
FACT-B	0–4	38	106.53	16.945	0.867
	5–10	44	102.73	21.949	

*DT* distress thermometer, *PBW* physical well being, *SWB* social/family well being, *EWB* emotional well being, *FWB* functional well being, *BCS* breast cancer subscale, *FACT-B* functional assessment of chronic illness therapy –breast.

### Index of alpha-, beta-diversity of different study groups in genus, species level

**Figure 1:**
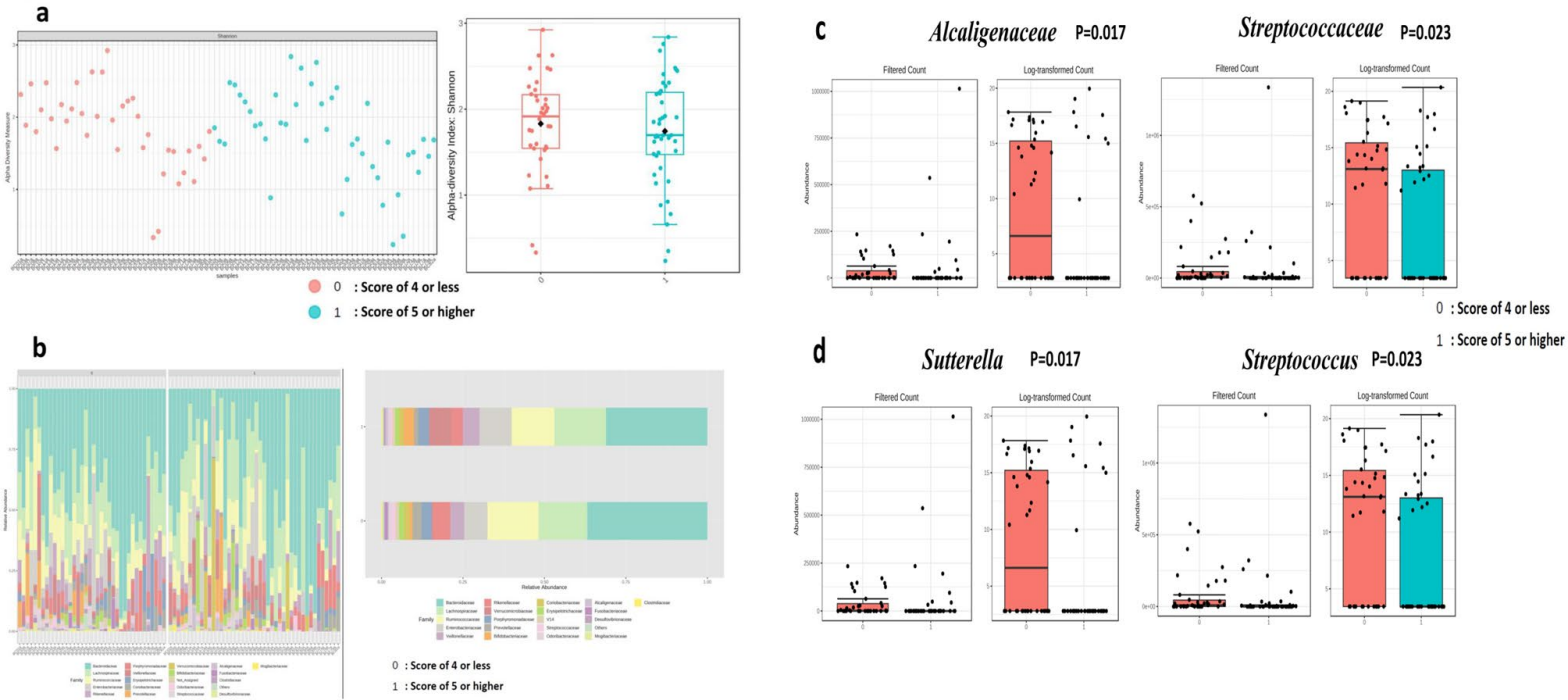
Identified potential bacteria biomarker in DT of breast cancer (Potential bacterial biomarkers in Family and Genus level with LEfSe analysis). Alpha-diversity by Shannon index and inter-quantile distribution indicate the richness and evenness divided with DT score (**1a**). Top 20 bacteria composition in genus level of each sample by percentage abundance ((**1b**), left). The most abundant genus of bacteria found in merge sample group were *Bacteroides* ((**1b**), right). In family level, *Alcaligenaceae* is significant in DT score less than 4 (p = 0.017, (**1c**) left), while *Streptococcaceae* is associated in DT score over 5 (p = 0.023, (**1c**) right). Further in genus level, A. Sutterella is a specific biomarker in DT score under 4 (p = 0.017, (**1d**) left). On the other hand, *S. Streptococcus* is a specific biomarker in DT score oner 5 (P = 0.023, (**1d**) right). Detailed LDA score list in Table 4. Using Linear discriminant analysis (LDA) Effect Size (LEfSe) under condition with p < 0.05, Log LDA score > 1.0. We identified some potential biomarker associated with DT score. However, we cannot identify more significant species in LEfSe due to bacteria diversity. (0: represent DT score less than 4; 1: represent DT score over than 5).

**Figure 2:**
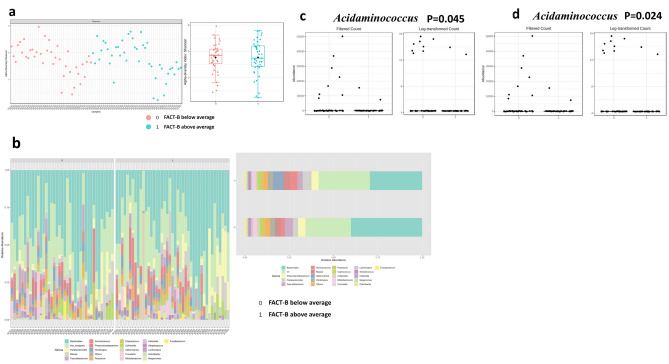
Identified potential bacteria biomarker in FACT-B of breast cancer. Alpha-diversity by Shannon index and inter-quantile distribution indicate the richness and evenness divided with FACT-B criteria (**2a**). Top 20 bacteria composition in genus level for each sample divided by FACT-B ((**2b**), left). Each label represents the percentage abundance of top 20 taxonomy. The most abundant genus of bacteria found in merged FACT-B group were *Bacteroides* ((**2b**), right). *Acidaminococcus* is a specific bacteria biomarker in FACT-G below average (p = 0.045, (**2c**)) and FACT-B below average (p = 0.024, (**2d**)). Detailed LDA score list in Table 5.

The index of alpha- and beta-diversity of different study groups in genus and species levels are presented in supplement Table 4 based on their DT scores, FACT subclass scores, depression and worry mentioned in DT problems, respectively. All the alpha-, beta-diversity parameters do not reach statistical significance. This may indicate that the differences between our samples of microbiota are small in this series. The alpha-diversity assessed by richness (Chao1, left box) and diversity (Shannon, right box) in the family level of DT score of 4 or less (0, pink dot color) and score of 5 or higher (1, blue dot) are shown in Fig. 1a. Barplots of the relative abundance of the 20 most abundant taxa identified to family level, found in DT score of 4 or less (0, lower row) and score of 5 or higher (1, upper row) are shown in Fig. 1b. Alpha-diversity by Shannon index and inter-quantile distribution indicate the richness and evenness divided with FACT-B criteria (Fig. 2a). Top 20 bacteria composition in genus level for each sample divided by FACT-B (Fig. 2b, left). Each label represents the percentage abundance of top 20 taxonomy. The most abundant genus of bacteria found in merged FACT-B group were Bacteroides (Fig. 2b, right).

**Table 4:** Significant top 5 relatively abundant bacterial taxa of DT groups in Family and Genus level**.**

	DT 0–4	DT 5–10	LDA score	FDR	p values
Family					
*Alcaligenaceae*	37,302	50,003	3.8	0.28134	0.017*
*Streptococcaceae*	74,404	54,195	− 4	0.28134	0.023*
*Erysipelotrichaceae*	306,340	141,720	− 4.92	0.9568	0.164
*Burkholderiaceae*	8520.6	11,606	3.19	0.9568	0.176
*V5*	62,940	67,250	3.33	0.9568	0.197
Genus					
*Sutterella*	37,302	50,003	3.8	0.39387	0.017*
*Streptococcus*	74,404	54,195	− 4	0.39387	0.023*
*Dorea*	41,974	23,079	− 3.98	0.61968	0.054
*Holdemania*	6583.6	3062.6	− 3.25	0.61968	0.071
*Lachnospira*	66,054	31,091	− 4.24	0.70652	0.102

*DT* distress thermometer, *LDA* latent Dirichlet allocation, *FDR* false discovery rate.

*p < 0.05.

### Significant top 5 relatively abundant bacterial taxa in different study groups

The top 5 bacterial taxa that are relatively abundant in different study groups were determined using LEfSe criteria, with a p value < 0.05 and log LDA score > 1. Table 5 and Supplement Tables 5 and 6 show the significant top 5 bacterial taxa in each group. Specifically, *Alcaligenaceae* (p = 0.017) at the family level and *Sutterella* (p = 0.017) at the genus level were found to be significantly more abundant in individuals with higher scores on the DT scale (Fig. 1c), while *Streptococcaceae* (p = 0.028) at the family level and Streptococcus (p = 0.023) at the genus level were significantly more abundant in individuals with lower scores on the DT scale (Fig. 1d). Moreover, *Christensenellaceae* (p = 0.008) and *Ruminococcaceae* (p = 0.025) at the family level, and* Faecalibacterium* (p = 0.014),* Coprococcus* (p = 0.046) at the genus level, and *Obeum* (p = 0.001), *Prausnitzii *(p = 0.014), and *Plebeius* (p = 0.0018) at the species level were significantly more abundant in breast cancer patients who reported having depression in the DT questionnaire. The relative abundance of *Eubacterium* (p = 0.019) at the family level and dolichum (p = 0.019) at the genus level were significantly higher in individuals who did not report having depression in the DT questionnaire.

**Table 5:** Significant top 5 relatively abundant bacterial taxa of FACT-G and FACT-B groups in Family and Genus level.

Family	FACT-G below average	FACT-G above average	LDA score	FDR	p values
*Clostridiaceae*	46,152	11,877	− 4.23	0.8018	0.048*
*Prevotellaceae*	143,960	219,760	4.58	0.8018	0.091
*Veillonellaceae*	335,740	314,110	− 4.03	0.8018	0.107
*Gemellaceae*	298.09	967.95	2.53	0.8018	0.158
*Mogibacteriaceae*	19,960	15,280	− 3.37	0.8018	0.160
Genus					
*Acidaminococcus*	4409.5	566.21	− 3.28	0.95114	0.045*
*Clostridium*	47,328	12,309	− 4.24	0.95114	0.051
*Granulicatella*	413.74	1585.2	2.77	0.97095	0.099
*Prevotella*	103,280	121,340	3.96	0.97095	0.136
*Holdemania*	5361.9	4014.9	− 2.83	0.97095	0.152

FACT-B group					
Family	FACT-B below average	FACT-B above average	LDA score	FDR	p values
*Alcaligenaceae*	21,127	64,473	4.34	0.80561	0.099
*Mogibacteriaceae*	20,608	15,019	− 3.45	0.80561	0.136
*Prevotellaceae*	155,030	204,420	4.39	0.80561	0.178
*Odoribacteraceae*	46,711	91,040	4.35	0.80561	0.203
*Ruminococcaceae*	1,309,000	1,045,200	− 5.12	0.80561	0.240
Genus					
*Acidaminococcus*	4748.7	526.7	− 3.32	0.83702	0.024*
*Anaerostipes*	45,465	16,553	− 4.16	0.83702	0.142
*Granulicatella*	445.56	1474.6	2.71	0.83702	0.172
*Ruminococcus*	274,200	357,390	4.62	0.83702	0.218
*Parabacteroides*	416,770	756,920	5.23	0.83702	0.222

*TOI* FACT-B Trial Outcome Index, (PWB score) + (FWB score) + (BCS score) = FACT-B TOI; FACT-G Total score = (PWB score) + (SWB score) + (EWB score) + (FWB score); FACT-B total score = (PWB score) + (SWB score) + (EWB score) + (FWB score) + (BCS score) (Please refer to supplement Table 1), *SD* standard deviation, *LDA* latent Dirichlet allocation, *FDR* false discovery rate.

*p < .05.

In the PWB above average group, there was a significantly higher relative abundance of *Alcaligenaceae* (p = 0.022) at the family level and *Sutterella* (p = 0.022) at the genus level. In the SWB group, the relative abundance of *Adlercreutzia* (p = 0.005) was significantly higher in individuals with below-average scores. In the EWB group, the relative abundance of *Carnobacteriacea*e (p = 0.044) at the family level and *Granulicatella* (p = 0.044) at the genus level were significantly higher in individuals with above-average scores. Conversely, in the EWB below average group, there was a significantly higher relative abundance of *Distasonis* (p= 0.032) and V2 (p = 0.037) at the species level. The relative abundance of *Prevotellaceae *(p = 0.045) at the family level and *Prevotella* (p = 0.045) at the genus level, and Copri (p = 0.045) at the species level were significantly higher in individuals with above-average scores of FWB group patients. Conversely, in the BCS below average group, there was a significantly higher relative abundance of *Lachnospiraceae* (p = 0.033) and *Pasteurellaceae* (p = 0.037) at the family level, and *Acidaminococcus* (p = 0.018) and* Haemophilus* (p = 0.037) at the genus level, and *Parainfluenzae* (p = 0.037) and *Catus* (p = 0.041) at the species level. The FACT-G below average group had a significantly higher relative abundance of *Clostridiaceae* (p = 0.048) at the family level, and *Acidaminococcus* (p = 0.045, Fig. 2c) at the genus level. The FACT-B below average group had a significantly higher relative abundance of *Acidaminococcus* (p = 0.024, Fig. 2d) at the genus level. Detailed LDA score list in Table 5.

## Discussion

To our knowledge, this is the first study to investigate the relationship between QOL, distress and the gut microbiome in newly diagnosed breast cancer patients. We sought to determine the relationship of distress and FACT-B different domain and fecal microbial composition among newly diagnosed breast cancer patients. Several associations between distress, FACT-B different domain and microbial taxa were observed among this sample of breast cancer patients.

From Table 2, treatment decisions, nervousness and worry are the most popular sources of distress that can be identified by patients using the Distress Thermometer (DT) in our series. Patients who rate their level of distress as 5 or higher on the DT and identify emotional concerns as a source of distress may benefit from further assessment or intervention to address these concerns. This may include referral to a mental health professional, such as a psychologist or psychiatrist, who can provide counseling or other forms of psychotherapy to help the patient manage their emotional distress. Other interventions that may be helpful for emotional concerns identified on the DT include support groups, relaxation techniques, and stress-reduction strategies, such as mindfulness meditation or yoga. Healthcare providers may also provide education and information about coping strategies and resources that are available to help patients manage emotional distress related to cancer and its treatment. Moreover, treatment decisions can be a significant source of anxiety and worry for many patients with breast cancer. Patients may feel overwhelmed by the complexity of treatment options, uncertain about the potential outcomes and side effects of different treatments. In clinical practice, healthcare providers should take steps to support patients in making informed decisions that are aligned with their goals and values. This may include providing clear and accurate information about treatment options, discussing the risks and benefits of different treatments, and engaging in shared decision-making with patients and their families^32,33^.

In this study, we observed significant differences in the abundance of certain bacterial families and genera in relation to the role of gut microbiota in distress and LOQ of newly diagnosed breast cancer patients. Specifically, *Alcaligenaceae* in the family level and *Sutterella* in the genus level were found to be significantly more abundant in individuals with higher scores on the DT scale, while individuals with below-average scores on the Functional Assessment of Cancer Therapy—General (FACT-G) scale had a significantly higher relative abundance of Clostridiaceae. Alcaligenaceae, a bacterial family within the gut microbiome, is implicated in a range of health and disease contexts. It shows associations with conditions such as inflammatory bowel diseases^34^, chronic kidney disease^35^, cholelithiasis^36^, thyroid cancer^37^, colorectal cancer^38^, esophageal squamous cell carcinoma^39^, and breast cancer^40^, suggesting potential roles in disease development or progression. The families *Pseudomonadaceae, Sphingomonadaceae, Alcaligenaceae, Ruminococcaceae*, and *Clostridia* were reported to be decreased in adjacent breast tissue compared with breast cancer tissue^41^. Additionally, the presence of Alcaligenaceae, a proinflammatory bacterial family, was found to be higher in depressed patients without anxiety compared to those with anxiety symptoms and the showed the proportion of Alcaligenaceae and Sutterella in the anxiety-negative depressed group was significantly higher than in the anxiety-positive group in first-episode depression of Chinese patients^42^. This suggests that the composition of the gut microbiota, including Alcaligenaceae, may influence the manifestation and severity of depression. However, more research is required to establish causality and understand the underlying mechanisms. *Sutterella*, belonging to Betaproteobacteria, are Gram-negative, non-spore-forming rods that grow in a microaerophilic atmosphere or under anaerobic conditions. Emerging research highlights the intricate relationship between the Sutterella and various aspects of health and disease, including irritable bowel disease, Crohn’s disease, autism spectrum disorder, Down syndrome and multiple sclerosis^43^, cancer therapy outcomes^44^, and sleep duration^45^. In the context of cancer therapy, particularly CAR-T cell therapy for hematologic malignancies, the presence of Sutterella in the gut microbiota has been associated with treatment responses and survival, emphasizing its potential role as a therapeutic target^44^. In contrast, studies related to autism spectrum disorder (ASD) have revealed differences in gut microbiota composition, including the presence of Sutterella, in children with ASD, suggesting a link between the microbiome and neurodevelopmental disorders. Conversely, lower abundance of *Sutterella* was been observed in people with depression^46^. Sutterella has demonstrated varying abundance levels in different psychiatric conditions, including lower levels in individuals with depression and higher levels in some children with autism^47^. This suggests its potential role in the intricate microbial-brain-gut axis. Additionally, Sutterella may possess immunomodulatory properties and contribute to Th-17 differentiation^43^. In a pilot study, lower relative abundance of Sutterella was consistently observed in adults with shorter sleep durations, suggesting a connection between this bacterium and sleep patterns^45^. Clostridiaceae plays a multifaceted role in health and disease. In menopause, it contributes to alterations in the gut microbiome, potentially affecting cardiometabolic health, with certain members like Clostridium lactatifermentans associated with protective effects against cardiovascular risk factors^48^. Additionally, Clostridiaceae, particularly Clostridium species, has been implicated in the gut microbiome of children with ASD, emphasizing its link to neurodevelopmental conditions^49^. The bidirectional relationship between stress, the hypothalamus-pituitary-adrenocortical axis, and the gut microbiome involves Clostridium species, potentially influencing overall well-being and distress^50^. Furthermore, emerging research suggests that Clostridiaceae bacteria may impact breast cancer outcomes by interacting with the immune system, highlighting their relevance in the context of cancer treatment and quality of life for survivors^51^.These findings suggest that the microbiome may play an important role in the development of distress and impacts of LOQ of newly diagnosed breast cancer patients.

The *Streptococcaceae* was significantly (p = 0.028) more abundant in individuals with lower scores on the DT scale in this study. Accompanied by inflammation, *Streptococcus mutans (S. mutans)*, an oral bacterium, invades endothelial cells (ECs) and disrupts their integrity, thereby promoting tumor cell extravasation and ultimately facilitating metastasis of breast cancer cells to the lungs^52^. The role of neurotransmitter imbalance, particularly insufficient levels of monoamine neurotransmitters like serotonin, dopamine, and norepinephrine, in contributing to emotional distress and depression. Serotonin, a key neurotransmitter in the brain-gut axis, is mainly synthesized in the gut by certain bacteria^53^. Various bacteria such as *Streptococcus spp., Enterococcus spp., Escherichia spp., Lactobacillus plantarum, Klebsiella pneumoniae, and Morganella morganii* were reported to have the ability to produce serotonin^54^. The high abundance of *Streptococcaceae* was observed in people with depression and the linkage between gut microbiota pattern and depression may be through the brain-gut microbiome axis^46^. One animal study revealed that a combination of living *Bifidobacterium, Lactobacillus* and *Streptococcus* may be used for treatment of anxiety^55^. But high abundance of *Streptococcaceae* was observed in people with depression^46^. It needs further study to define the role of *Streptococcaceae* in the distress and QOL in newly diagnosed breast cancer patients and evaluate its potential interventions of biomarker.

The primary limitation of the Distress Thermometer (DT) in this study lies in its potential inadequacy for assessing the complex relationship between psychological distress and different problem list variables, such as pain, in newly diagnosed breast cancer patients. For example, while the study aims to screen for distress in this population using the DT scale, it faces challenges in capturing the nuances of pain experiences and their psychological impact. The DT's single-item nature remains subjective and may not sufficiently differentiate between different sources and origins of pain, making it less suitable for assessing pain-related distress comprehensively. Moreover, the study's diverse breast cancer patient population and the potential variations in distress of newly diagnosed cancer patients highlight the need for a more tailored and multidimensional assessment approach. Therefore, while the DT is recommended routine screening for distress in all cancer patients since1999 by the National Comprehensive Cancer Network (NCCN)^56^. The DT was developed as a simple tool to effectively screen for symptoms of distress and offers a user-friendly screening tool^57^, its limitations in addressing the multifaceted nature of different problems, such as pain, and distress in newly diagnosed breast cancer patients should be recognized, and supplementary assessments or tools may be necessary for a more in-depth understanding of this complex relationship. We would like to clarify that our study primarily aimed to explore the microbiome's potential links with depressive tendencies as measured by the DT scale, rather than to establish a direct causative relationship.

In conclusion, this prospective study defines the relationships among QOL, stress and gut microbiota in newly diagnosed breast cancer patients and provides many useful information to find potential interventions of biomarker or probiotics for decreasing stress and for improving quality of life in breast cancer patients.

### Supplementary Information

Supplementary Information.
