# Supplementary material for: Unraveling the connections between gut microbiota, stress, and quality of life for holistic care in newly diagnosed breast cancer patients
Source: Sci Rep. 2023 Oct 20;13:17916. doi: 10.1038/s41598-023-45123-1 (PMC10589294; doi:10.1038/s41598-023-45123-1)
Supplement: Supplementary file 1 — Supplementary Information. [file 41598_2023_45123_MOESM1_ESM.docx]

**Title: Unraveling the Connections Between Gut Microbiota, Stress, and Quality of Life for Holistic Care in Newly Diagnosed Breast Cancer Patients**

**Authors: Chi-Chan Lee^1^, Horng-Woei Yang^3^, Chih-Ju Liu^4,5^, Fang Lee^1^, Wen-Ching Ko^1^, Yuan-Ching Chang^1^, Po-Sheng Yang*^1,2^**

1. Department of General Surgery, MacKay Memorial Hospital, Taipei, Taiwan
2. Department of Medicine, MacKay Medical College, New Taipei, Taiwan
3. Division of Molecular Medicine, Department of Medical Research, MacKay Memorial Hospital, Taipei, Taiwan
4. Department of Nursing, MacKay Memorial Hospital, Taipei, Taiwan
5. Department of Nursing, MacKay Medical College, New Taipei, Taiwan

Corresponding author: Po-Sheng Yang, email address: psyang@mmc.edu.tw

**Supplement Table 1. Functional Assessment of Chronic Illness Therapy (FACT)-Breast questionnaire and Scoring Guidelines (Version 4)**

**Below is a list of statements that other people with your illness have said are important. Please circle or mark one number per line to indicate your response as it applies to the past 7 days.**

|  | **PHYSICAL WELL-BEING** | **Not at all** | **A little bit** | **Some-what** | **Quitea bit** | **Very much** |
| --- | --- | --- | --- | --- | --- | --- |
|  |  |  |  |  |  |  |
| **GP1** | **I have a lack of energy** | **0** | **1** | **2** | **3** | **4** |
| **GP2** | **I have nausea** | **0** | **1** | **2** | **3** | **4** |
| **GP3** | **Because of my physical condition, I have trouble meeting the needs of my family** | **0** | **1** | **2** | **3** | **4** |
| **GP4** | **I have pain** | **0** | **1** | **2** | **3** | **4** |
| **GP5** | **I am bothered by side effects of treatment** | **0** | **1** | **2** | **3** | **4** |
| **GP6** | **I feel ill** | **0** | **1** | **2** | **3** | **4** |
| **GP7** | **I am forced to spend time in bed** | **0** | **1** | **2** | **3** | **4** |
|  | | | | | | |
|  | **SOCIAL/FAMILY WELL-BEING** | **Not at all** | **A little bit** | **Some-what** | **Quitea bit** | **Very much** |
|  |  |  |  |  |  |  |
| **GS1** | **I feel close to my friends** | **0** | **1** | **2** | **3** | **4** |
| **GS2** | **I get emotional support from my family** | **0** | **1** | **2** | **3** | **4** |
| **GS3** | **I get support from my friends** | **0** | **1** | **2** | **3** | **4** |
| **GS4** | **My family has accepted my illness** | **0** | **1** | **2** | **3** | **4** |
| **GS5** | **I am satisfied with family communication about my illness** | **0** | **1** | **2** | **3** | **4** |
| **GS6** | **I feel close to my partner (or the person who is my main support)** | **0** | **1** | **2** | **3** | **4** |
| **Q1** | ***Regardless of your current level of sexual activity, please answer the following question. If you prefer not to answer it, please mark this box and go to the next section.*** |  |  |  |  |  |
| **GS7** | **I am satisfied with my sex life** | **0** | **1** | **2** | **3** | **4** |

**Please circle or mark one number per line to indicate your response as it applies to the past 7 days.**

|  | **EMOTIONAL WELL-BEING** | **Not at all** | **A little bit** | **Some-what** | **Quitea bit** | **Very much** |
| --- | --- | --- | --- | --- | --- | --- |
|  |  |  |  |  |  |  |
| **GE1** | **I feel sad** | **0** | **1** | **2** | **3** | **4** |
| **GE2** | **I am satisfied with how I am coping with my illness** | **0** | **1** | **2** | **3** | **4** |
| **GE3** | **I am losing hope in the fight against my illness** | **0** | **1** | **2** | **3** | **4** |
| **GE4** | **I feel nervous** | **0** | **1** | **2** | **3** | **4** |
| **GE5** | **I worry about dying** | **0** | **1** | **2** | **3** | **4** |
| **GE6** | **I worry that my condition will get worse** | **0** | **1** | **2** | **3** | **4** |

|  | **FUNCTIONAL WELL-BEING** | **Not at all** | **A little bit** | **Some-what** | **Quitea bit** | **Very much** |
| --- | --- | --- | --- | --- | --- | --- |
|  |  |  |  |  |  |  |
| **GF1** | **I am able to work (include work at home)** | **0** | **1** | **2** | **3** | **4** |
| **GF2** | **My work (include work at home) is fulfilling** | **0** | **1** | **2** | **3** | **4** |
| **GF3** | **I am able to enjoy life** | **0** | **1** | **2** | **3** | **4** |
| **GF4** | **I have accepted my illness** | **0** | **1** | **2** | **3** | **4** |
| **GF5** | **I am sleeping well** | **0** | **1** | **2** | **3** | **4** |
| **GF6** | **I am enjoying the things I usually do for fun** | **0** | **1** | **2** | **3** | **4** |
| **GF7** | **I am content with the quality of my life right now** | **0** | **1** | **2** | **3** | **4** |

**Please circle or mark one number per line to indicate your response as it applies to the past 7 days.**

|  | **ADDITIONAL CONCERNS** | **Not at all** | **A little bit** | **Some-what** | **Quitea bit** | **Very much** |
| --- | --- | --- | --- | --- | --- | --- |
|  |  |  |  |  |  |  |
| **B1** | **I have been short of breath** | **0** | **1** | **2** | **3** | **4** |
| **B2** | **I am self-conscious about the way I dress** | **0** | **1** | **2** | **3** | **4** |
| **B3** | **One or both of my arms are swollen or tender** | **0** | **1** | **2** | **3** | **4** |
| **B4** | **I feel sexually attractive** | **0** | **1** | **2** | **3** | **4** |
| **B5** | **I am bothered by hair loss** | **0** | **1** | **2** | **3** | **4** |
| **B6** | **I worry that other members of my family might someday get the same illness I have** | **0** | **1** | **2** | **3** | **4** |
| **B7** | **I worry about the effect of stress on my illness** | **0** | **1** | **2** | **3** | **4** |
| **B8** | **I am bothered by a change in weight** | **0** | **1** | **2** | **3** | **4** |
| **B9** | **I am able to feel like a woman** | **0** | **1** | **2** | **3** | **4** |
| **P2** | **I have certain parts of my body where I experience pain** | **0** | **1** | **2** | **3** | **4** |

**FACT-B Scoring Guidelines** (Version 4) – Page 1

Instructions:* 1. Record answers in "item response" column. If missing, mark with an X

2. Perform reversals as indicated, and sum individual items to obtain a score.

3. Multiply the sum of the item scores by the number of items in the subscale, then divide by the

number of items answered. This produces the subscale score.

4. Add subscale scores to derive total scores (TOI, FACT-G & FACT-B).

5. **The higher the score, the better the QOL.**

**Subscale Item Code Reverse item? Item response Item Score**

**PHYSICAL** GP1 4 - ________ =________

**WELL‑BEING** GP2 4 - ________ =________

**(PWB)** GP3 4 - ________ =________

GP4 4 - ________ =________

*Score range:* 0-28

GP5 4 - ________ =________

GP6 4 - ________ =________

GP7 4 - ________ =________

***Sum individual item scores:*** ________

***Multiply by 7:*** ________

***Divide by number of items answered:*** **________**=**PWB subscale score**

**SOCIAL/FAMILY** GS1 0 + ________ =________

**WELL‑BEING** GS2 0 + ________ =________

**(SWB)** GS3 0 + ________ =________

GS4 0 + ________ =________

GS5 0 + ________ =________

*Score range:* 0-28

GS6 0 + ________ =________

GS7 0 + ________ =________

***Sum individual item scores:*** ________

***Multiply by 7:*** ________

***Divide by number of items answered:*** **________*=*SWB subscale score**

**EMOTIONAL** GE1 4 - ________ =________

**WELL‑BEING** GE2 0 + ________ =________

**(EWB)** GE3 4 - ________ =________

GE4 4 - ________ =________

*Score range:* 0-24

GE5 4 - ________ =________

GE6 4 - ________ =________

***Sum individual item scores:*** ________

***Multiply by 6:*** ________

***Divide by number of items answered:*** **________*=*EWB subscale score**

**FUNCTIONAL**  GF1 0 + ________ =________

**WELL-BEING**  GF2 0 + ________ =________

**(FWB)** GF3 0 + ________ =________

GF4 0 + ________ =________

GF5 0 + ________ =________

*Score range:* 0-28

GF6 0 + ________ =________

GF7 0 + ________ =________

***Sum individual item scores:*** ________

***Multiply by 7:*** ________

***Divide by number of items answered:*** **________**=**FWB subscale score**

**FACT-B Scoring Guidelines** (Version 4) – Page 2

**Subscale Item Code Reverse item? Item response Item Score**

**BREAST**  B1 4 - ________ =________

**CANCER** B2 4 - ________ =________

**SUBSCALE** B3 4 - ________ =________

**(BCS)** B4 0 + ________ =________

B5 4 - ________ =________

*Score range:* 0-40

B6 4 - ________ =________

B7 4 - ________ =________

B8 4 - ________ =________

B9 0 + ________ =________

P2 4 - ________ =________

***Sum individual item scores:***________

***Multiply by 10:*** ________

***Divide by number of items answered:*** **________*=*BC Subscale score**

**To derive a FACT-B Trial Outcome Index (TOI):**

*Score range:* 0-96

__________ + __________ + __________ =**________**=**FACT-B TOI**

(**PWB** score) (**FWB** score) (**BCS** score)

**To Derive a FACT-G total score:**

*Score range:* 0-108

__________ + __________ + __________ + __________=**________**=**FACT-G Total score**

(**PWB** score) (**SWB** score) (**EWB** score) (**FWB** score)

**To Derive a FACT-B total score:**

*Score range:* 0-148

_________ + __________ + __________ + __________ + __________ =**________**=**FACT-B Total score**

(**PWB** score) (**SWB** score) (**EWB** score) (**FWB** score) (**BCS** score)

**Supplement Table 2. Comparison of DT score and socioeconomic, stage and treatment modality**

|  |  | DT Score 0-4 | | DT Score 5-10 | | P value |
| --- | --- | --- | --- | --- | --- | --- |
|  |  | n | % | n | % |  |
| Age | <= 50 years | 10 | 31.3 | 22 | 68.9 | 0.041* |
|  | > 51 years | 28 | 56.0 | 22 | 44.0 |  |
| Sleep | Normal | 25 | 48.1 | 27 | 51.9 | 0.427 |
|  | Insomnia/ Easy to wake up | 13 | 43.3 | 17 | 56.7 |  |
| Economic status | Advantage | 31 | 48.8 | 33 | 51.6 | 0.328 |
|  | Disadvantage | 7 | 38.9 | 11 | 61.1 |  |
| Education | Below bachelor's degree | 10 | 71.4 | 4 | 28.6 | 0.045* |
|  | Above bachelor's degree | 28 | 41.2 | 40 | 58.8 |  |
| Marital status | Unmarried | 4 | 26.7 | 11 | 73.3 | 0.211 |
|  | Married | 25 | 49.0 | 26 | 51.0 |  |
|  | Divorced/ Widowed | 9 | 56.3 | 7 | 43.8 |  |
| FACT-B score | Below 104.5 | 15 | 30.5 | 24 | 61.5 | 0.127 |
|  | Above 104.6 | 23 | 53.5 | 20 | 46.5 |  |
| Stage | 0 | 0 | 0.0 | 2 | 100.0 | 0.448 |
|  | I | 9 | 47.4 | 10 | 52.6 |  |
|  | II | 23 | 47.9 | 25 | 52.1 |  |
|  | III | 5 | 55.6 | 4 | 44.4 |  |
|  | IV | 1 | 25.0 | 3 | 75.0 |  |
| Chemotherapy | No | 6 | 54.5 | 5 | 45.5 | 0.395 |
|  | Yes | 32 | 45.1 | 39 | 54.9 |  |
| Radiotherapy | No | 21 | 43.8 | 27 | 56.3 | 0.369 |
|  | Yes | 17 | 50.0 | 17 | 50.0 |  |
| Hormone therapy | No | 17 | 51.5 | 16 | 48.5 | 0.293 |
|  | Yes | 21 | 42.9 | 28 | 57.1 |  |

Abbreviations DT: Distress Thermometer ; FACT-B: Functional Assessment of Chronic Illness Therapy -Breast; *p<.05

**Supplement Table 3. Comparison of DT and Lefestyle variants**

|  |  | DT score 0-4 | | DT score 5-10 | | P value |
| --- | --- | --- | --- | --- | --- | --- |
|  |  | n | % | n | % |  |
| Alcohol consumption | No | 34 | 48.6 | 36 | 51.4 | 0.367 |
|  | Yes | 4 | 33.3 | 8 | 66.7 |  |
| Use of gastroenterology medications | No | 32 | 47.8 | 35 | 52.2 | 0.776 |
|  | Yes | 6 | 40.0 | 9 | 60.0 |  |
| Use of antibiotics | No | 26 | 48.1 | 28 | 51.9 | 0.816 |
|  | Yes | 12 | 42.9 | 16 | 57.1 |  |
| History of gastroenteritis | No | 37 | 45.7 | 44 | 54.3 | 0.463 |
|  | Yes | 1 | 100.0 | 0 | 0.0 |  |
| History of irritable bowel disease | No | 37 | 46.3 | 43 | 53.8 | 1.000 |
|  | Yes | 1 | 50.0 | 1 | 50.0 |  |
| History of inflammatory bowel disease | No | 36 | 45.6 | 43 | 54.4 | 0.594 |
|  | Yes | 2 | 66.7 | 1 | 33.3 |  |
| Constipation or chronic diarrhea | No | 32 | 49.2 | 33 | 50.6 | 0.415 |
|  | Yes | 6 | 35.3 | 11 | 64.7 |  |
| Bloody stools | No | 37 | 46.3 | 43 | 53.8 | 1.000 |
|  | Yes | 1 | 50.0 | 1 | 50.0 |  |
| History of colon polyp | No | 37 | 45.7 | 44 | 54.3 | 0.463 |
|  | Yes | 1 | 100.0 | 0 | 0.0 |  |

Abbreviations DT: Distress Thermometer

**Supplement Table 4. Shannon Alpha-diversity and Beta-diversity of different study groups in Genus, Species level**

| **Genus alpha-diversity** | | | **Species alpha-diversity** | | |  |
| --- | --- | --- | --- | --- | --- | --- |
|  | P value | T-test |  | P value | T-test |  |
| **DT** | 0.506 | 0.669 |  | 0.319 | 1.002 |  |
| **PWB** | 0.538 | -0.620 |  | 0.839 | -0.205 |  |
| **SWB** | 0.827 | 0.220 |  | 0.526 | 0.638 |  |
| **EWB** | 0.802 | 0.251 |  | 0.860 | 0.177 |  |
| **FWB** | 0.833 | -0.212 |  | 0.755 | 0.313 |  |
| **BCS** | 0.455 | 0.751 |  | 0.282 | 1.084 |  |
| **FACT-G** | 0.921 | -0.099 |  | 0.717 | 0.364 |  |
| **FACT-B** | 0.966 | -0.043 |  | 0.744 | 0.327 |  |
| **Depression in DT** | 0.264 | -1.155 |  | 0.240 | -1.228 |  |
| **Worry in DT** | 0.733 | 0.343 |  | 0.446 | 0.767 |  |
|  |  |  |  |  |  |  |
| **Genus Beta-diversity** | | | | **Species Beta-diversity** | | |
|  | F value | r-square | P value | F value | r-square | P value |
| **DT** | 1.444 | 0.018 | 0.165 | 0.535 | 0.007 | 0.857 |
| **PWB** | 0.506 | 0.006 | 0.854 | 0.604 | 0.007 | 0.763 |
| **SWB** | 0.499 | 0.006 | 0.853 | 1.467 | 0.018 | 0.128 |
| **EWB** | 0.850 | 0.011 | 0.537 | 0.511 | 0.006 | 0.866 |
| **FWB** | 1.372 | 0.017 | 0.198 | 1.492 | 0.018 | 0.135 |
| **BCS** | 0.942 | 0.012 | 0.468 | 0.960 | 0.012 | 0.455 |
| **FACT-G** | 0.713 | 0.009 | 0.689 | 0.934 | 0.012 | 0.494 |
| **FACT-B** | 0.717 | 0.009 | 0.692 | 0.666 | 0.008 | 0.752 |
| **Depression in DT** | 0.766 | 0.009 | 0.611 | 1.869 | 0.023 | 0.065 |
| **Worry in DT** | 0.557 | 0.007 | 0.814 | 0.892 | 0.011 | 0.519 |

Abbreviations and Definition: DT: Distress Thermometer ; PBW: Physical well being; SWB: Social/Family well being; EWB: Emotional well being; FWB: Functional well being; BCS: Breast cancer subscale; FACT-B: Functional Assessment of Chronic Illness Therapy -Breast ; FACT-G Total score = (PWB score) + (SWB score) + (EWB score) + (FWB score); FACT-B total score= (PWB score) + (SWB score) + (EWB score) + (FWB score) + (BCS score) (Please refer to supplement table 1)

**Supplement Table 5. Significant top 5 relatively abundant bacterial taxa of FACT-B subclass in Family, Genus and Species level**

| **PWB Group** |  |  |  |  |  |
| --- | --- | --- | --- | --- | --- |
| **Family** | PWB below average | PWB above average | LDA score | FDR | P values |
| *Alcaligenaceae* | 17458 | 64187 | -4.37 | 0.544 | 0.022* |
| *Streptococcaceae* | 27007 | 88657 | -4.49 | 0.757 | 0.111 |
| *Turicibacteraceae* | 443.65 | 4526.9 | -3.31 | 0.757 | 0.123 |
| *Ruminococcaceae* | 1467700 | 1083600 | 5.28 | 0.757 | 0.165 |
| *Fusobacteriaceae* | 3853.8 | 78126 | -4.57 | 0.757 | 0.176 |
| **Genus** |  |  |  |  |  |
| *Sutterella* | 17458 | 64187 | -4.37 | 0.784 | 0.022* |
| *Streptococcus* | 27007 | 88657 | -4.49 | 0.888 | 0.111 |
| *Turicibacter* | 443.65 | 4526.9 | -3.31 | 0.888 | 0.123 |
| *Eggerthella* | 10675 | 7611.1 | 3.19 | 0.888 | 0.149 |
| *Fusobacterium* | 3853.8 | 78126 | -4.57 | 0.888 | 0.176 |
|  |  |  |  |  |  |
| **SWB group** |  |  |  |  |  |
| **Family** | SWB below average | SWB above average | LDA score | FDR | P values |
| *Gemellaceae* | 63.901 | 965.83 | -2.66 | 0.658 | 0.050 |
| *Mogibacteriaceae* | 28083 | 11352 | 3.92 | 0.658 | 0.053 |
| *Veillonellaceae* | 321010 | 327730 | -3.53 | 0.711 | 0.095 |
| *Fusobacteriaceae* | 65486 | 14898 | 4.4 | 0.711 | 0.114 |
| *Verrucomicrobiaceae* | 92104 | 209460 | -4.77 | 0.843 | 0.169 |
| **Genus** |  |  |  |  |  |
| *Adlercreutzia* | 4876 | 1385.6 | 3.24 | 0.180 | 0.005* |
| Fusobacterium | 104960 | 15215 | 4.65 | 0.900 | 0.114 |
| Phascolarctobacterium | 294390 | 253090 | 4.31 | 0.900 | 0.121 |
| Roseburia | 236680 | 152500 | 4.62 | 0.900 | 0.129 |
| Akkermansia | 92097 | 218690 | -4.8 | 0.900 | 0.169 |
|  |  |  |  |  |  |
| **EWB group** |  |  |  |  |  |
| **Family** | EWB below average | EWB above average | LDA score | FDR | P values |
| *Carnobacteriaceae* | 441.38 | 1649.1 | 2.78 | 0.783 | 0.044* |
| *Alcaligenaceae* | 26932 | 67985 | 4.31 | 0.783 | 0.060 |
| *Lachnospiraceae* | 1615200 | 1406200 | -5.02 | 0.793 | 0.104 |
| *Veillonellaceae* | 339450 | 283930 | -4.44 | 0.793 | 0.122 |
| *Desulfovibrionaceae* | 24867 | 18715 | -3.49 | 0.834 | 0.206 |
| **Genus** |  |  |  |  |  |
| *Granulicatella* | 441.38 | 1649.1 | 2.78 | 0.939 | 0.044* |
| *Sutterella* | 26932 | 67985 | 4.31 | 0.939 | 0.060 |
| *Acidaminococcus* | 3448.8 | 972.92 | -3.09 | 0.939 | 0.104 |
| *Oscillospira* | 244020 | 352830 | 4.74 | 0.939 | 0.107 |
| *Coprococcus* | 246480 | 129340 | -4.77 | 0.951 | 0.174 |
| **Species** |  |  |  |  |  |
| *distasonis* | 26582 | 11933 | -3.86 | 0.428 | 0.032* |
| *V2* | 7213600 | 6539200 | -5.53 | 0.428 | 0.037* |
| *producta* | 1709 | 20053 | 3.96 | 0.986 | 0.142 |
| *torques* | 36231 | 111590 | 4.58 | 0.986 | 0.234 |
| *caccae* | 140900 | 115000 | -4.11 | 0.986 | 0.285 |
|  |  |  |  |  |  |
| **FWB group** |  |  |  |  |  |
| **Family** | FWB below average | FWB above average | LDA score | FDR | P values |
| *Prevotellaceae* | 19498 | 320350 | -5.18 | 0.450 | 0.045* |
| *Alcaligenaceae* | 18772 | 65522 | -4.37 | 0.450 | 0.056 |
| *Ruminococcaceae* | 1418900 | 956360 | 5.36 | 0.450 | 0.058 |
| *Gemellaceae* | 132.59 | 1050 | -2.66 | 0.450 | 0.072 |
| *Veillonellaceae* | 325220 | 325160 | 1.44 | 0.585 | 0.117 |
| **Genus** |  |  |  |  |  |
| *Prevotella* | 19618 | 318500 | -5.17 | 0.773 | 0.045* |
| *Butyricimonas* | 9784.9 | 34977 | -4.1 | 0.773 | 0.079 |
| *Anaerostipes* | 45608 | 21115 | 4.09 | 0.773 | 0.111 |
| *Holdemania* | 5595.4 | 4042.9 | 2.89 | 0.773 | 0.136 |
| *Faecalibacterium* | 388020 | 277660 | 4.74 | 0.773 | 0.141 |
| **Species** |  |  |  |  |  |
| *copri* | 19618 | 318500 | -5.17 | 0.681 | 0.045* |
| *ovatus* | 425350 | 391460 | 4.23 | 0.681 | 0.113 |
| *prausnitzii* | 388020 | 277660 | 4.74 | 0.681 | 0.141 |
| *aerofaciens* | 119720 | 232650 | -4.75 | 0.681 | 0.148 |
| *producta* | 6888.7 | 12358 | -3.44 | 0.681 | 0.167 |
|  |  |  |  |  |  |
| **BCS group** |  |  |  |  |  |
| **Fmily** | BCS below average | BCS above average | LDA score | FDR | P values |
| *Lachnospiraceae* | 1716100 | 1355600 | -5.26 | 0.462 | 0.033* |
| *Pasteurellaceae* | 15813 | 5898.2 | -3.7 | 0.462 | 0.037* |
| *Verrucomicrobiaceae* | 73617 | 245640 | 4.93 | 0.624 | 0.128 |
| *Streptococcaceae* | 21615 | 98950 | 4.59 | 0.624 | 0.140 |
| *Coriobacteriaceae* | 260890 | 143740 | -4.77 | 0.624 | 0.144 |
| **Genus** |  |  |  |  |  |
| *Acidaminococcus* | 4780.2 | 586.91 | -3.32 | 0.528 | 0.018* |
| *Haemophilus* | 15813 | 5898.2 | -3.7 | 0.528 | 0.037* |
| *Blautia* | 548130 | 368170 | -4.95 | 0.528 | 0.053 |
| *Coprococcus* | 304500 | 105970 | -5 | 0.528 | 0.057 |
| *Akkermansia* | 73617 | 245640 | 4.93 | 0.629 | 0.128 |
| **Species** |  |  |  |  |  |
| *parainfluenzae* | 15813 | 5898.2 | -3.7 | 0.439 | 0.037* |
| *catus* | 10159 | 427.75 | -3.69 | 0.439 | 0.041* |
| *obeum* | 21487 | 9010.5 | -3.8 | 0.439 | 0.075 |
| *ovatus* | 456910 | 366260 | -4.66 | 0.439 | 0.097 |
| *muciniphila* | 73617 | 245640 | 4.93 | 0.439 | 0.128 |

Abbreviations and Definition: PWB: Physical well being; SWB: Social/Family well being; EWB: Emotional well being; FWB: Functional well being; BCS: Breast cancer subscale; FACT-B: Functional Assessment of Chronic Illness Therapy -Breast ; FACT-G Total score = (PWB score) + (SWB score) + (EWB score) + (FWB score); FACT-B total score= (PWB score) + (SWB score) + (EWB score) + (FWB score) + (BCS score) (Please refer to supplement table 1) ; LDA: Latent Dirichlet Allocation; FDR: False Discovery Rate; *p<.05

**Supplement Table 6. Significant top 5 relatively abundant bacterial taxa of DT subgroups in Family, Genus and Species level**

| **Family** | Without depression in DT | With depression in DT | LDA score | FDR | P values |
| --- | --- | --- | --- | --- | --- |
| *Christensenellaceae* | 226.55 | 1194.8 | 2.69 | 0.204 | 0.008* |
| *Ruminococcaceae* | 1014500 | 1531100 | 5.41 | 0.323 | 0.025* |
| *Burkholderiaceae* | 9983.3 | 12739 | 3.14 | 0.788 | 0.113 |
| *Clostridiaceae* | 26478 | 50363 | 4.08 | 0.788 | 0.157 |
| *Mogibacteriaceae* | 14961 | 19290 | 3.34 | 0.788 | 0.158 |
| **Genus** |  |  |  |  |  |
| *Faecalibacterium* | 297790 | 502160 | 5.01 | 0.338 | 0.014* |
| *Eubacterium* | 9413.9 | 0 | -3.67 | 0.338 | 0.019* |
| *Coprococcus* | 130610 | 339570 | 5.02 | 0.553 | 0.046* |
| *Roseburia* | 153280 | 380480 | 5.06 | 0.605 | 0.067 |
| *Burkholderia* | 9983.3 | 12739 | 3.14 | 0.718 | 0.113 |
| **Species** |  |  |  |  |  |
| *obeum* | 9914.1 | 42719 | 4.21 | 0.113 | 0.011* |
| *prausnitzii* | 297790 | 502160 | 5.01 | 0.113 | 0.014* |
| *plebeius* | 247210 | 401220 | 4.89 | 0.113 | 0.018* |
| *dolichum* | 9413.9 | 0 | -3.67 | 0.113 | 0.019* |
| *catus* | 4354 | 6200.9 | 2.97 | 0.365 | 0.076 |
|  |  |  |  |  |  |
| **Mentioned Worry in DT Group** | |  |  |  |  |
| **Family** | Without worry in DT | With worry in DT | LDA score | FDR | P values |
| *V1* | 105970 | 40608 | -4.51 | 0.578 | 0.044* |
| *Alcaligenaceae* | 7183.6 | 33290 | 4.12 | 0.578 | 0.044* |
| *Pasteurellaceae* | 17069 | 60.963 | -3.93 | 0.638 | 0.074 |
| *Mogibacteriaceae* | 20086 | 8204.8 | -3.77 | 0.728 | 0.115 |
| *Clostridiaceae* | 27944 | 33311 | 3.43 | 0.728 | 0.168 |
| **Genus** |  |  |  |  |  |
| *Sutterella* | 7183.6 | 33290 | 4.12 | 0.703 | 0.044* |
| *Haemophilus* | 17069 | 60.963 | -3.93 | 0.703 | 0.074 |
| *Holdemania* | 5179.6 | 4101 | -2.73 | 0.703 | 0.138 |
| *Clostridium* | 27944 | 33311 | 3.43 | 0.703 | 0.168 |
| *Collinsella* | 208490 | 103900 | -4.72 | 0.703 | 0.177 |

Abbreviations DT: Distress Thermometer ; LDA: Latent Dirichlet Allocation; FDR: False Discovery Rate; *p<.05


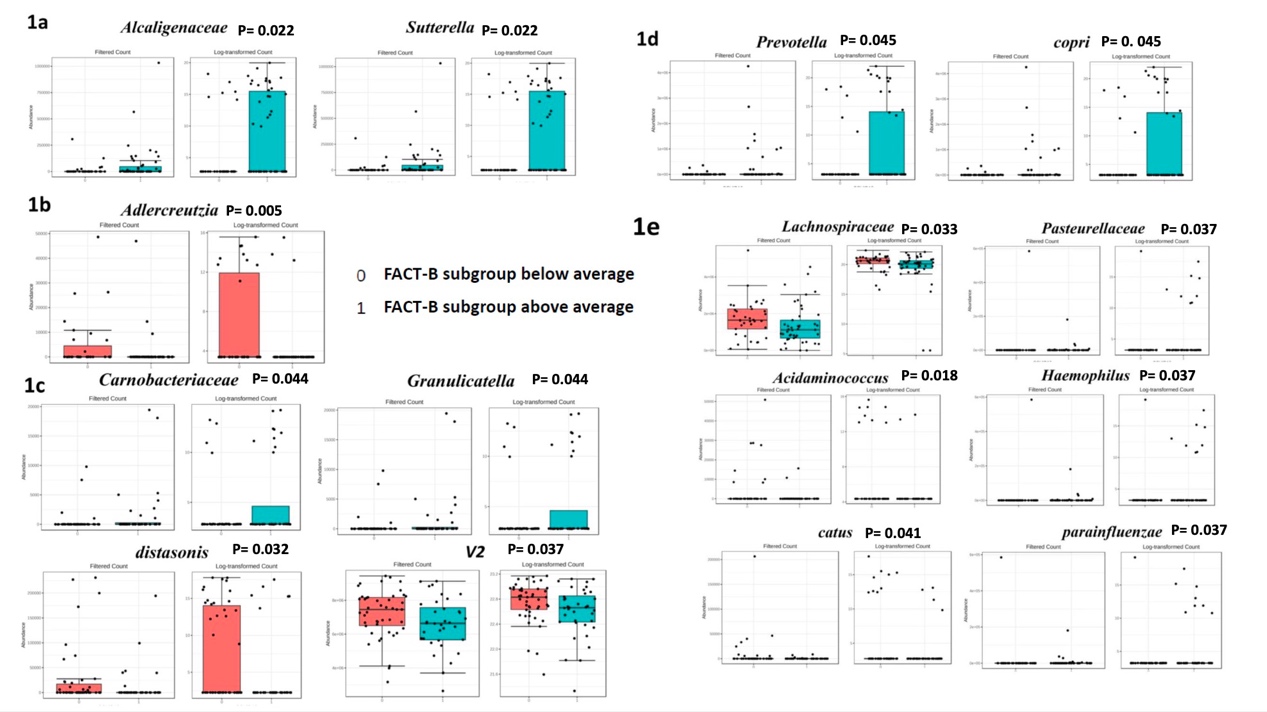


**Supplementary Figure 1. Bacterial Biomarkers Influence Various Wellbeing Domains in Breast Cancer Patients.** The FACT-B assessment tool incorporates various evaluation criteria encompassing physical wellbeing (PWB, 1a), social/family wellbeing (SWB, 1b), emotional wellbeing (EWB, 1c), functional wellbeing (FWB, 1d), and a domain specific to breast cancer (BCS, 1e). Potential bacterial biomarkers were determined using LEfSe, along with their corresponding grouping criteria. Notably, in the realm of PWB, the *Alcaligenaceae Sutterella* was found to be associated with a PWB score exceeding 23.8 (P=0.022, 1a). In the context of SWB, the genus *Adlercreutzia* was identified with a score below 19.5 (P=0.005, 1b). Regarding EWB, the *Carnobacteriaceae Granulicatella* exhibited significance in elevating EWB scores above 18.6 (P=0.044, 1c upper), whereas at the species level, *distasonis* and *V2* were associated with EWB scores below 18.6 (P=0.031, P=0.037, 1c lower). In terms of FWB, *Prevotella Copri* dominated and correlated with FWB scores surpassing 18.5 (P=0.045, 1d). In the BCS domain, *Pasteurellaceae Haemophilus parainfluenzae* was identified, resulting in BCS scores below 24.1 (P=0.037, 1e right), and at various taxonomic levels (family *Lachnospiraceae*, genus *Acidaminococcus*, and species *catus*), similar associations were noted with BCS scores under 24.1 (P=0.033, 0.018, and 0.041, 1e left). Detailed LDA score information is available in Supplementary Table 5.
